# Electronic Structures of Chlorophyll *a* Investigated by Nitrogen K‑Edge X‑ray Absorption Spectroscopy under a Radiation-Induced Effect

**DOI:** 10.1021/acs.jpca.5c07907

**Published:** 2025-12-16

**Authors:** Fumitoshi Kumaki, Shota Tsuru, Shohei Yamashita, Jun-ichi Adachi, Masanari Nagasaka

**Affiliations:** † Photon Factory, Institute of Materials Structure Science, High Energy Accelerator Research Organization, Tsukuba, Ibaraki 305-0801, Japan; ‡ Department of Chemistry, Keio University, Yokohama 223-8522, Japan; § 530386 RIKEN Center for Computational Science, RIKEN, Kobe 650-0047, Japan; ∥ Graduate Institute for Advanced Studies, SOKENDAI, Tsukuba, Ibaraki 305-0801, Japan; ⊥ Institute for Molecular Science and Graduate Institute for Advanced Studies, SOKENDAI, Okazaki 444-8585, Japan

## Abstract

The electronic structures of high-purity solid chlorophyll *a* were investigated using nitrogen K-edge X-ray absorption spectroscopy. The C=N π* peaks of the chlorins exhibited characteristic split profiles, which changed progressively with successive scans owing to radiation-induced effects. Inner-shell quantum chemical calculations for structural models of chlorophyll *a* with different phytol chain lengths assigned the electronic structures of the C=N π* peaks and confirmed that the intensity changes of the C=N π* peaks are caused by cleaving the phytol chain. The influence of the vibration of the phytol chain to the C=N π* peaks of the chlorins was also discussed.

## Graphical abstract

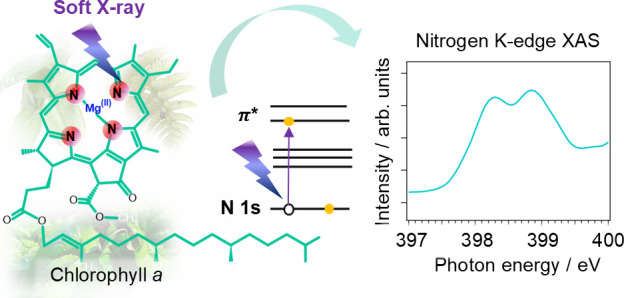

## 1 Introduction

Chlorophyll is a major photosynthetic pigment and is used in photodynamic therapy^1^ and dye-sensitized solar cells^2^ owing to its ability to absorb visible light. The photosynthesis of terrestrial plants functions with complementary uses of chlorophyll *a* (Chl-a) and chlorophyll *b*, where solar light is effectively absorbed over the wide wavelength range of visible light.^3–5^ The hydrophobicity of the phytol chain in Chl-a plays a role in distributing Chl-a uniformly in lipids to achieve highly efficient photosynthetic reactions. Meanwhile, the role of the phytol group has not been fully understood in energy and electron transfers during Chl-a photosynthesis.^6–11^ The photochemistry of Chl-a has been investigated using several methods such as vibrational spectroscopy, ultraviolet–visible spectroscopy, nuclear magnetic resonance, and circular dichroism measurements.^12–18^ The lifetimes of the photoexcited states in Chl-a were also investigated using ultrafast spectroscopy:^19,20^ The lifetime of the lowest singlet (S_1_) state is on the order of a few nanoseconds to several tens of nanoseconds, and that of the lowest triplet (T_1_) state is on the order of several hundreds of microseconds. These lifetimes are much longer than those of copper chlorophyllin, a derivative of Chl-a without a phytol chain, where the lifetime of the S_1_ state is 22 ps and that of the T_1_ state has not been observed.^21^

Soft X-ray absorption spectroscopy (XAS) is a method that can clarify the electronic and vibrational structures. The electronic structures of Chl-a can be investigated from both the central metal and ligand sides using XAS, where the central magnesium is measured at the Mg K-edge and the chlorin ring is measured at the C, N, and O K-edges. Previous studies have measured the XAS spectra of Chl-a at the Mg and C K-edges.^22–24^ The Mg K-edge XAS spectrum of Chl-a is less sensitive to electronic structural changes in chlorin rings with different side chains, such as the phytol chain. In the C K-edge XAS spectrum, separating the contribution of the phytol chain from that of the chlorins is difficult because Chl-a consists of numerous carbon atoms. Meanwhile, the N K-edge XAS measurement is effective for investigating the electronic structures of Chl-a owing to metal–ligand delocalization.^25–28^ Charge transfer processes in metal complexes have been extensively studied using N K-edge XAS.^29–31^ The differences in electronic structures and spin multiplicities between iron and cobalt porphyrin complexes were investigated using N K-edge XAS.^28^ The photorelaxation process of the iron phenanthroline complex was investigated from the ligand side using time-resolved N K-edge XAS with a time scale of several tens of picoseconds.^32^

In this study, the electronic structures of Chl-a were investigated by N K-edge XAS of high-purity solid Chl-a. The C=N π* peaks of chlorins changed with successive scans owing to radiation-induced effects. Radiation-induced effects have drawn attention for the application of XAS to organic molecules and biomolecules, where the chemical bonds of organic molecules, polymers, adenosine triphosphate, deoxyribose, and DNAs are broken by radiation.^33–41^ Inner-shell calculations of Chl-a with different chain lengths were conducted to assign the electronic structures of the C=N π* peaks and the spectral changes caused by radiation-induced effects were discussed.

## 2 Methods

### 2.1 XAS Experiment

N K-edge XAS experiments were performed in the multibunch operation mode with a ring current of 450 mA at the soft X-ray beamline BL-19B of the Photon Factory, Institute of Materials Structure Science, High Energy Accelerator Research Organization (KEK-PF).^42^ The XAS spectra of the Chl-a samples were measured in the partial fluorescence yield mode using a silicon drift detector, which was normalized with the intensities of incident soft X-rays, and were obtained with three successive scans at the same sample position. Chl-a powder with a purity of 98.6% was purchased from Fuji S. L. I. and was uniformly dispersed using a mortar. As shown in Section S1 of the Supporting Information, Chl-a powder was spread especially thinly on indium plates in a copper sample holder to minimize self-absorption of X-ray fluorescence, which was set at room temperature under an ultrahigh vacuum condition below 4 × 10^–5^ Pa. Each XAS spectrum was obtained with the scan time of 400 s, where the X-ray fluorescence was measured from 390 to 430 eV with an energy step of 0.1 eV and an accumulation time of 1.0 s. The beam size of soft X-rays was 200 and 50 μm at the horizontal and vertical axes, respectively.^43^ The photon flux was adjusted to 7 × 10^10^ photons/s at 400 eV by controlling the width of the exit slit, which was precisely measured using a photodiode detector. Therefore, the photon fluence of one spectrum was estimated to be 2.8 × 10^15^ photons/mm^2^. The photon energies were precisely calibrated from the N K-edge XAS spectra of titanium nitrides.^42^

### 2.2 Inner-Shell Calculation

For each system, the geometry was optimized at the density-functional-theory (DFT) level and the inner-shell spectrum was calculated by time-dependent DFT (TDDFT)^44^ using the program package ORCA 6.0.1.^45,46^ The CAM-B3LYP functional^47^ and def2-TZVPP basis set^48^ were adopted throughout the present study for the structural optimizations using DFT and inner-shell calculations using TDDFT. The number of roots demanded in the inner-shell calculations was 20. The isosurface was set to 0.01 for visualizing the Kohn–Sham orbitals. The excitation energies in the inner-shell calculations were broadened using a Gaussian profile with a full width at half-maximum of 0.5 eV. The oscillator strengths of excitation energies are plotted 10-fold. The inner-shell spectrum calculated for each system was shifted by +12.05 eV, considering the energetic position of the first peak in the N K-edge XAS spectrum of tetraphenylporphyrins, as shown in Section S5 of the Supporting Information.

## 3 Results and Discussion

### 3.1 XAS Spectra of Chl-a under a Radiation-Induced Effect

Figure 1a shows the N K-edge XAS spectra of solid Chl-a with three successive scans. The peaks near 399 eV are assigned to the transition of N 1s electrons to the C=N π* orbitals of chlorins, whose energy regions are shown in Figure 1b. Two C=N π* peaks with the energetic positions of (a) 398.20 eV and (b) 398.91 eV were observed. These C=N π* peaks are derived from the LUMO and LUMO+1 orbitals from nonequivalent nitrogen atoms in chlorin rings including different substituents and phytol chains, whose assignments are precisely described in Section 3.2. Notably, the N K-edge XAS spectra of metal complexes did not show significant spectral changes due to aggregation.^28^ Therefore, the aggregation of Chl-a does not affect the N K-edge XAS spectrum, whereas aggregation affects the visible absorption spectrum.

**Figure 1:**
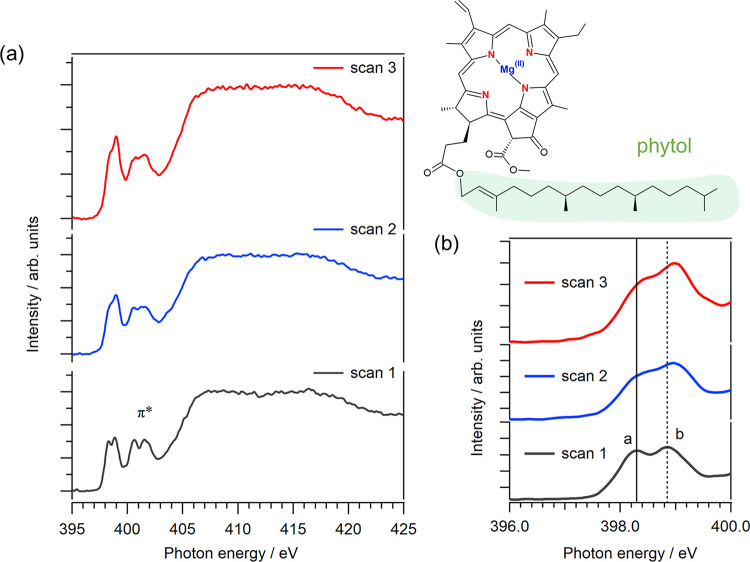
(a) N K-edge XAS spectra of solid Chl-a with three successive scans at the same sample position. (b) Expansion of the C=N π* peaks in the N K-edge XAS spectra of solid Chl-a. The solid and dashed lines represent the energetic positions of peaks (a) and (b) at the first scan, respectively.

Table 1 shows the energetic positions of the two C=N π* peaks, (a) and (b), from the N K-edge XAS spectra at three successive scans from the fitting procedure, the details of which are described in Section S2 of the Supporting Information. The intensity of peak (b) is almost equal to that of peak (a) in the first scan, confirming that the intensity ratio of peak (b) to peak (a) is 1.3. By continuing successive scans, the intensities of peak (a) decrease compared to those of peak (b): the intensity ratio (b)/(a) in the second scan is 2.1, and that in the third scan is 2.3. The spectral changes in the C=N π* peaks are related to the radiation-induced effects such as the cleavage of side chains, including the phytol chain.

**Table 1:** Energetic Positions of Peaks (a) and (b) in the N K-Edge XAS Spectra of Chl-a with Three Successive Scans, as Shown in Figure 1 ^a^

	energy (a)/eV	energy (b)/eV	ratio (b)/(a)
scan 1	398.20 ± 0.01	398.91 ± 0.01	1.3
scan 2	398.20 ± 0.03	398.95 ± 0.02	2.1
scan 3	398.20 ± 0.05	398.96 ± 0.03	2.3

a The intensity ratios of peak (b) to peak (a) are also shown.

### 3.2 Peak Assignments of Chl-a Using Inner-Shell Calculation

Figure 2 shows the calculated N K-edge inner-shell spectrum of Chl-a together with the experimental spectrum from the first scan. The first C=N π* peak is assigned to the transition of N 1s electrons to the LUMO orbitals, and the second peak is assigned to the transition to the LUMO+1 orbitals. Because the chlorin ring includes four nonequivalent nitrogen atoms, each N atom shows different energetic positions of the LUMO peaks. The LUMO peak at the highest energy side shows the energetic position of 398.65 eV, which is higher by 0.64 eV than that of the lowest energy side (398.01 eV). The highest LUMO peak is derived from the nitrogen atom closest to the phytol group and is nearly the same energetic position from the lowest LUMO+1 peak (399.08 eV). Thus, the first peak of the C=N π* peaks was assigned to the transitions to the LUMO orbitals from the 1s orbitals of the nitrogen atoms except for the nitrogen atom closest to the phytol group. The second peak was assigned to the transition to the LUMO orbital from the 1s orbital of the nitrogen atom closest to the phytol group and the transitions to the LUMO+1 orbitals. Note that the highest LUMO+1 peak, which comes from the transition of the 1s orbital of the nitrogen atom closest to the phytol group to the LUMO+1 orbital, shows the energetic position of 399.72 eV and is higher than the other LUMO+1 peaks. The highest energy excitations of the LUMO and LUMO+1 orbitals were derived from the N 1s Kohn–Sham orbital energy (−391.68 eV), which was lower than those of the other nitrogen atoms (−391.12, −391.04, and −391.01 eV). It is because the C–C bonds in the five-membered ring with the phytol chain are saturated.

**Figure 2:**
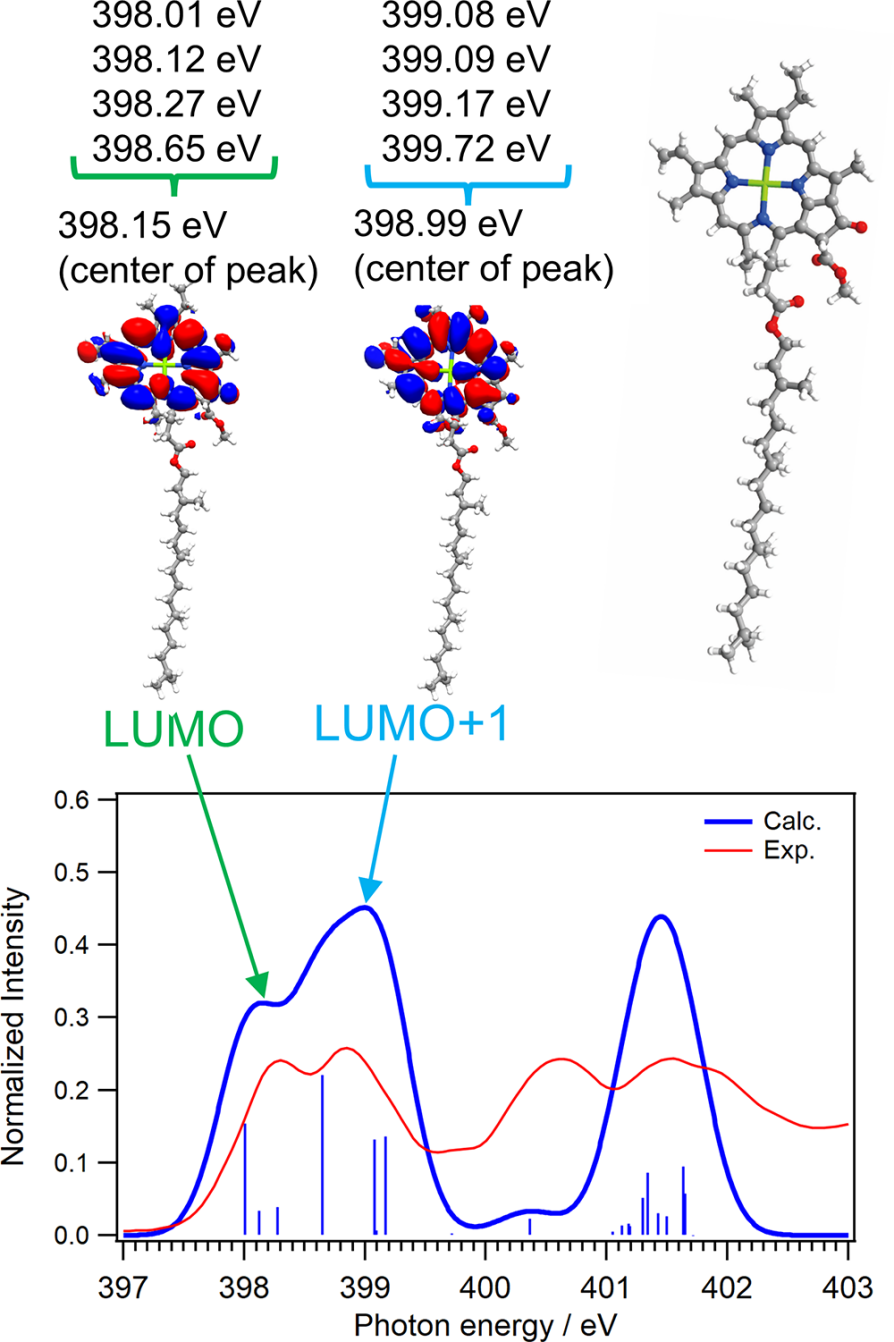
Calculated N K-edge inner-shell spectrum of Chl-a, together with the experimental spectrum at the first scan. The C=N π* peak consists of two peaks, which are assigned to the transition of N 1s electrons to the LUMO and LUMO+1 orbitals shown in the inset. The assignments of each N 1s electron to LUMO and LUMO+1 orbitals were also described.

In contrast, the higher peaks in the energy region from 400 to 403 eV were not reproduced by the TDDFT calculations. This energy region includes several electronic excitation processes from the N 1s electrons to LUMO+2, LUMO+3, and higher unoccupied orbitals, as discussed in Section S3 of the Supporting Information. Reproducing the higher peaks by TDDFT calculations is difficult because this energy region includes excitation to charge transfer states, such as electronic transitions to orbitals with Rydberg character, as well as those distributed outside the chlorins. Therefore, this study focuses on the C=N π* peaks in the energy region below 400 eV.

### 3.3 Inner-Shell Calculations of Chl-a with Different Chain Lengths

Figure 3 shows the calculated N K-edge inner-shell spectra of Chl-a with different chain structures to discuss the spectral changes in the C=N π* peaks with radiation-induced effects. The model structure of Chl-a with an incomplete chain excluding a phytol group was formed by breaking the ester bond between the propionic acid and phytol chains, followed by the addition of a hydroxy group. The model structure of Chl-a without a complete chain was formed by cleaving the bond in the five-membered ring and then adding a hydrogen atom.

**Figure 3:**
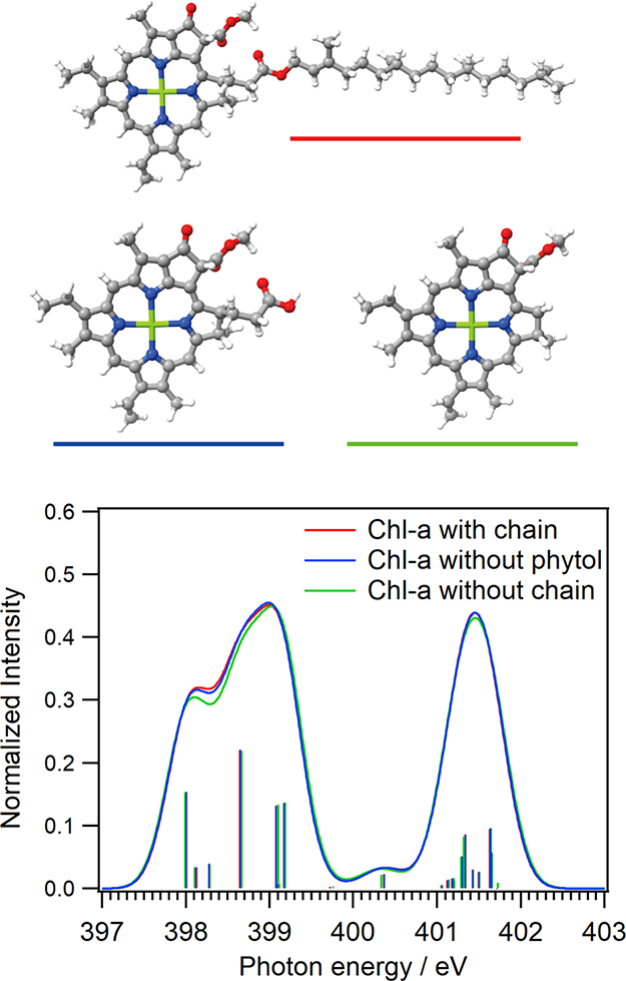
Calculated N K-edge inner-shell spectra of Chl-a with different chain structures: (red) complete Chl-a with a whole chain; (blue) Chl-a with an incomplete chain excluding a phytol group; and (green) Chl-a without a whole chain. These molecular structures are shown in the inset.

The intensities of the first peaks in the C=N π* peaks are slightly decreased by removing the phytol group and are decreased more clearly by removing the whole chain. It means that the spectral changes of the C=N π* peaks under the radiation-induced effects shown in Figure 1 are caused by removing the side chain including the phytol group. Note that the spectral changes in the inner-shell spectra of Chl-a without chains are smaller than those observed in the XAS spectra under the radiation-induced effects. The spectral changes of the C=N π* peaks are not only caused by the mixtures of the C=N π* orbitals of chlorins with the unoccupied orbitals of the phytol chain. The additional effect of the phytol chain is discussed in Section 3.4.

The radiation-induced desorption of the Mg^2+^ ion from Chl-a is discussed using the inner-shell calculations. In the inner-shell spectrum of the metal-free Chl-a anion, the LUMO and LUMO+1 peaks show lower energy shifts of approximately 1 eV compared with those of Chl-a, as described in Section S4 of the Supporting Information. This indicates that the Mg^2+^ ion was not desorbed from Chl-a under the present radiation-induced conditions. The calculated N K-edge inner-shell spectrum of metal-free tetraphenylporphyrins, which is analogous to the protonated Mg-desorbing Chl-a, also confirmed that the desorption of the Mg^2+^ ion does not occur with radiation-induced effects, as discussed in Section S5 of the Supporting Information. Because Mg^2+^ ions were not desorbed from Chl-a with the radiation-induced effects, the chlorin rings are not broken in the present condition. Therefore, the present study considered the removal of the phytol chain, which is the major side chain. Considering the removal of other side chains complicates the mechanism of the radiation-induced effects, which will be the subject of further studies.

### 3.4 Discussion about Vibration Effect of Phytol Chain

Inner-shell calculations confirm that the spectral changes of the C=N π* peaks are caused by the removal of the phytol chain. However, the spectral changes in the inner-shell spectra of Chl-a without chains are smaller than that observed in the XAS spectra under the radiation-induced effects, even though TDDFT with the CAM-B3LYP functional should reproduce intensities and relative energetic positions of the C=N π* peaks. One possibility for the additional spectral changes of the C=N π* peaks with induced-radiation effects are the changes in the vibrational fine structure in Chl-a, which might affect the fine structure of the XAS spectrum by vibrational excitations induced by the electronic transitions to the LUMO. The XAS spectra, which include vibrational fine structures, were used to investigate the molecular vibrations.^49,50^ Discrete vibrational fine structures exist at the higher-energy side of the main peaks in the XAS spectra with high energy resolution.^51,52^ As schematically described in Figure 4a, the complete Chl-a with a phytol chain may exhibit prominent anharmonicity in bending motions and many internal twist vibrational modes in the phytol chain. The vibrational satellites of the first C=N π* peaks [peak (a)] may lie close to the main peak and contribute to the intensity of peak (a) because the anharmonicity makes satellite peak distances narrow and these vibrational modes show low frequencies. When Chl-a without a phytol chain is formed by radiation-induced effects, as shown in Figure 4b, the vibrational satellites due to the twist vibrational modes disappear and the intensity of peak (a) decreases. Instead, the intensity of peak (b) becomes higher than that of peak (a) because the vibrational satellites of peak (a) due to vibrational modes with high frequencies may merge with those of peak (b). Notably, vibrational excitations were not considered in the present inner-shell calculations because this study focuses on not the determination of the individual vibrational fine structures but the discussion about the changes of asymmetric C=N π* features by removing the phytol chain. Inner-shell calculations considering vibration excitation for Chl-a are cumbersome because of the computational expenses and the subject of further studies.

**Figure 4:**
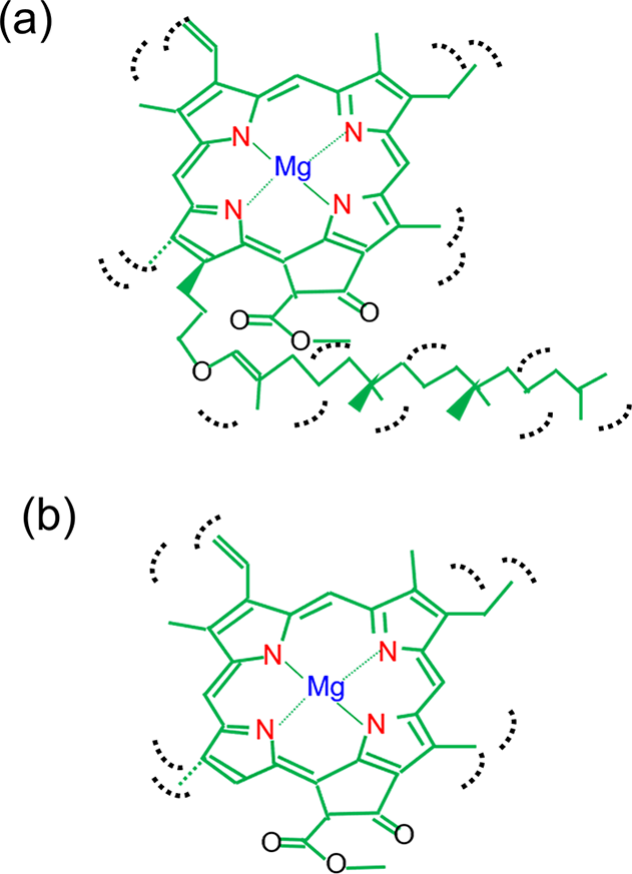
Schematics for molecular vibrations of Chl-a (a) with a phytol chain and (b) without a phytol chain.

## 4 Conclusions

The electronic structures of high-purity solid Chl-a were investigated using N K-edge XAS. The C=N π* peaks originating from the four nitrogen atoms in the chlorin ring consist of two peaks: (a) and (b). Compared with the intensity of the first peak (a), that of the second peak (b) increases with successive XAS scans owing to radiation-induced effects. Inner-shell calculations revealed that the two C=N π* peaks are derived from the excitation of nonequivalent nitrogen core electrons to the LUMO and LUMO+1 orbitals. The spectral changes in the C=N π* peaks of the XAS spectra under the radiation-induced effects were reproduced by the removal of the phytol chains from Chl-a in the inner-shell calculations. The additional spectral changes may be caused by changes in the vibrational fine structures owing to the loss of the phytol chain. The present study proposes that the C=N π* peaks of the chlorins are influenced by the unoccupied orbitals of the phytol chains with additional vibrational structures and will be useful for the further study of the charge transfer processes in Chl-a during photosynthetic reactions. The N K-edge XAS measurements of Chl-a in natural environments, such as solutions and proteins, will be realized using the liquid cell for XAS in a transmission mode.^28,53,54^ The energy transfers in the photosynthetic reactions of Chl-a in proteins can be investigated using time-resolved N K-edge XAS on the order of several tens of picoseconds.^32^

## Supplementary Material



## Data Availability

The data underlying this study are openly available in Figshare at DOI: 10.6084/m9.figshare.29604638.^55^

## Notes

Photo of the sample environment; fitting analysis of N K-edge XAS spectra; discussion of higher peaks in inner-shell calculation of chlorophyll *a*; calculated N K-edge inner-shell spectrum of metal-free chlorophyll *a* anion; and calculated N K-edge inner-shell spectrum of metal-free tetraphenylporphyrin (PDF)
