# Supplementary material for: Electronic Structures of Chlorophyll a Investigated by Nitrogen K‑Edge X‑ray Absorption Spectroscopy under a Radiation-Induced Effect
Source: J Phys Chem A. 2025 Dec 16;130(1):222–8. doi: 10.1021/acs.jpca.5c07907 (PMC12794170; doi:10.1021/acs.jpca.5c07907)
Supplement: Supplementary file 1 [file jp5c07907_si_001.pdf]

Supporting Information

Electronic Structures of Chlorophyll *a* Investigated by Nitrogen K-Edge X-ray Absorption Spectroscopy under a Radiation-Induced Effect

Fumitoshi Kumaki,[*,1,2] Shota Tsuru,[3] Shohei Yamashita,[1,4] Jun-ichi Adachi,[1,4] and Masanari Nagasaka [*,5]

[1] *Photon Factory, Institute of Materials Structure Science, High Energy Accelerator Research Organization, Tsukuba, Ibaraki 305-0801, Japan*

[2] *Department of Chemistry, Keio University, Yokohama 223-8522, Japan*

[3] *RIKEN Center for Computational Science, RIKEN, Kobe 650-0047, Japan*

[4] *Graduate Institute for Advanced Studies, SOKENDAI, Tsukuba, Ibaraki 305-0801, Japan*

[5] *Institute for Molecular Science and Graduate Institute for Advanced Studies, SOKENDAI, Okazaki 444-8585, Japan*

*Corresponding Authors
E-mail: kumakif@post.kek.jp (F. Kumaki)
E-mail: nagasaka@ims.ac.jp (M. Nagasaka)

Table of Contents **Page**

S1. Photo of sample environment S2
S2. Fitting analysis of N K-edge XAS spectra S2
S3. Discussion of higher peaks in inner-shell calculation of chlorophyll *a* S3
S4. Calculated N K-edge inner-shell spectrum of metal-free chlorophyll *a* anion S4
S5. Calculated N K-edge inner-shell spectrum of metal-free tetraphenylporphyrin S5

References S7

S1. Photo of sample environment

Figure S1 shows the photograph of chlorophyll *a* (Chl-a) powder spread especially thinly on indium plates for the N K-edge X-ray absorption spectroscopy (XAS) measurements in the fluorescence yield mode.

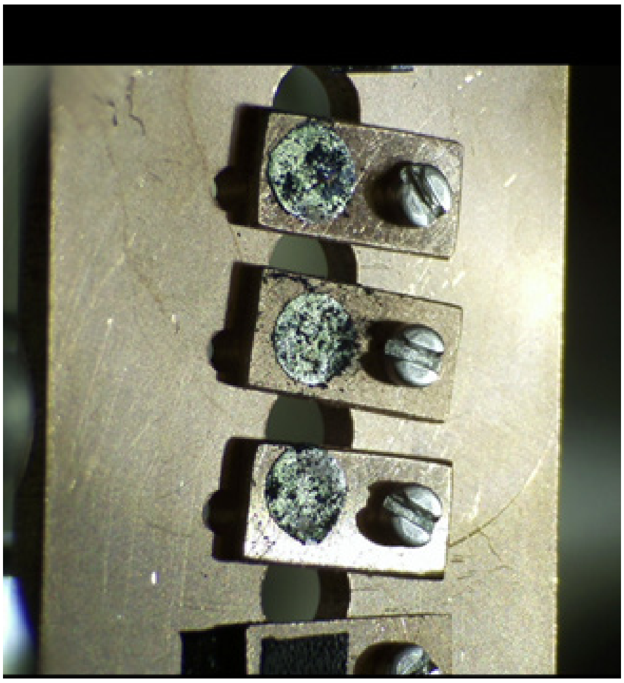

Figure S1. Photograph of Chl-a samples for the XAS measurements in the fluorescence yield mode. The Chl-a powder was spread especially thinly on indium plates in a copper sample holder.

S2. Fitting analysis of N K-edge XAS spectra

Figure S2 shows the fitting analysis for the N K-edge XAS spectra of Chl-a with three successive scans. The region of the C=N π* peaks were fitted with three gaussian peaks. The XAS spectra of Chl-a were changed during the successive scans owing to the radiation-induced effect. The intensities and energetic positions of the C=N π* peaks obtained by the fitting analysis are shown in Table 1.

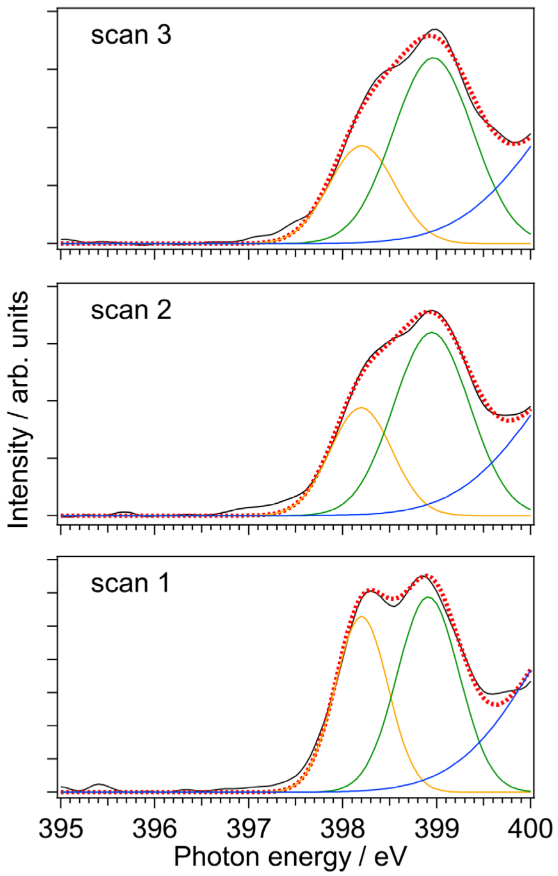

Figure S2. Fitting analysis for the N K-edge XAS spectra of solid Chl-a with three successive scans. The energy regions of the C=N π* peaks were fitted with three gaussian peaks.

S3. Discussion of higher peaks in inner-shell calculation of chlorophyll *a*

The higher energy peaks in the energy region from 400 to 403 eV were not reproduced by the inner-shell calculation using the time-dependent density functional theory (TDDFT), as shown in Fig. 2. Figure S3 shows the orbitals lying above LUMO+1, which contribute to the roots with excitation energies above 400 eV. Some orbitals show the Rydberg characters, and the densities of some orbitals are distributed at the phytol chain. These visualized orbitals indicate that the roots with excitation energies above 400 eV have charge-transfer characters, which are usually difficult to describe with TDDFT. This fact may explain the disagreement between the measured and calculated inner-shell spectra above 400 eV.

It is known that the quantitative evaluation of the excited states with charge-transfer character needs at least untruncated description of double-electronic excitations such as those in the equation-of-motion coupled cluster singles and doubles (EOM-CCSD).[1] Nevertheless, EOM-CCSD is practically not applicable to Chl-a because of the computational cost, i.e. the scaling factor of EOM-CCSD is O[(N^6)] where N is the number of basis set while that of TDDFT is O[(N^4)] in cases of hybrid functionals including CAM-B3LYP. Considering this computational limitation, fine structures of the region above 400 eV are not discussed in the present study.

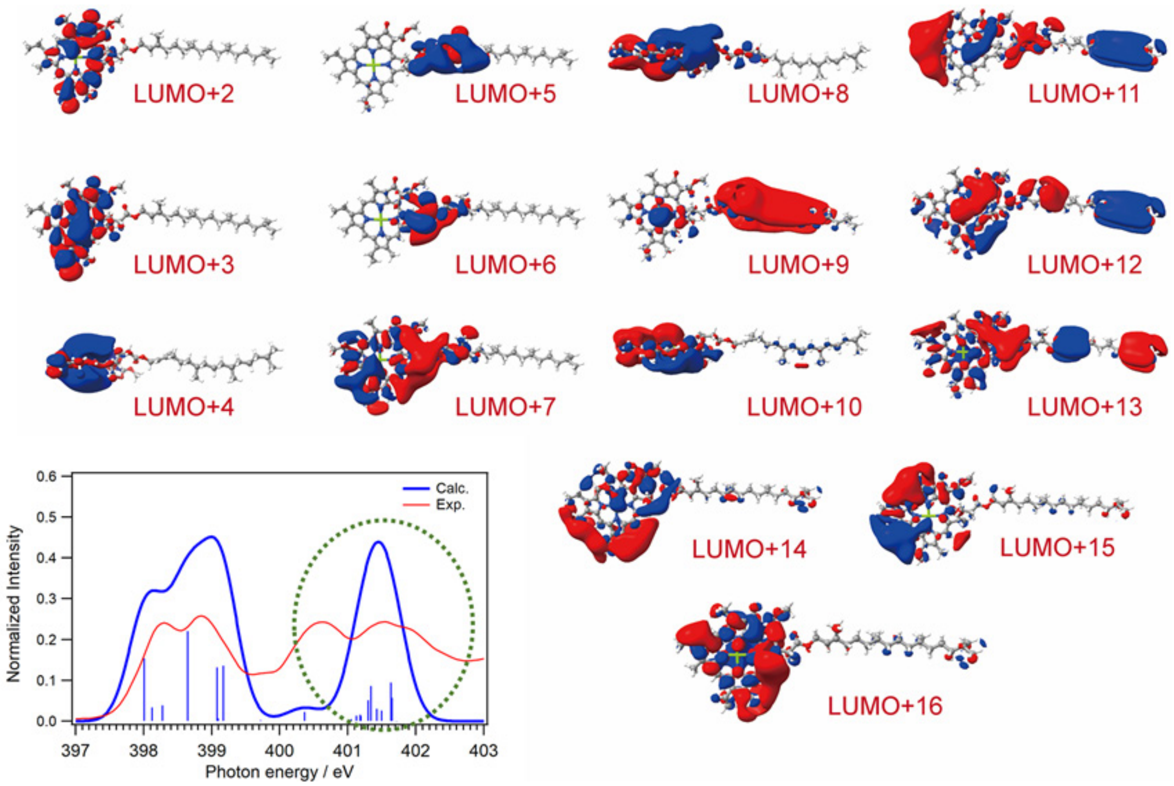

Figure S3. The higher unoccupied orbitals for the excitation of N 1s electrons in Chl-a, which has difficulties reproducing the higher peaks indicated by the circle in the experimental XAS spectrum.

S4. Calculated N K-edge inner-shell spectrum of metal-free chlorophyll *a* anion

Figure S4 shows the N K-edge inner-shell spectrum calculated for Chl-a anion without Mg^{2+} ion. The energetic positions of the first and second peaks were 397.02 eV and 398.04 eV, respectively. These peaks are assigned to the transition of the N 1s electrons to the LUMO and LUMO+1 orbitals, and show red shifts by ~1 eV from the C=N π* peaks in the N K-edge XAS spectrum of Chl-a. Such red shifts of the LUMO and LUMO+1 peaks were not observed in the present measurements. This fact supports the interpretation that the Mg^{2+} ion was not desorbed from Chl-a with the radiation-induced effect during the successive scans of the N K-edge XAS measurements.

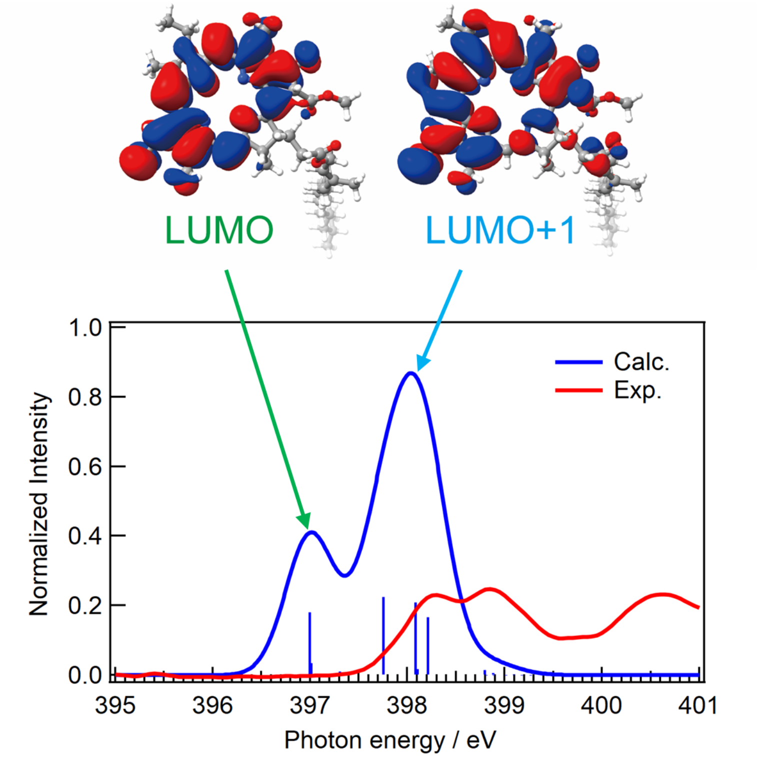

Figure S4. The calculated N K-edge inner-shell spectrum of Chl-a anion without Mg^{2+} ion, together with the experimental spectrum of Chl-a at the first scan.

S5. Calculated N K-edge inner-shell spectrum of metal-free tetraphenylporphyrin

Figure S5 shows the calculated N K-edge inner-shell spectrum of tetraphenylporphyrin, together with the N K-edge XAS spectrum of solid tetraphenylporphyrin. The spectral shapes of the inner-shell calculations were nearly same as those obtained by the XAS experiment. This XAS spectrum was consistent with the previous studies,[2] where the peak at 397.9 eV is assigned to the C–N=C group and that at 400.1 eV is assigned to the C–(NH)–C group. The calculated spectrum consists of the LUMO peak at 397.9 eV, the LUMO+1 peak at 399.8 eV, and the higher π^* peaks at 401.3 eV. Although most of the excited states at 400 – 401 eV are dark states in the TDDFT calculation at the optimized geometry, where the molecule is highly symmetric, these excited states would become optically bright owing to the conformational fluctuation of solid tetraphenylporphyrin.

In the calculated N K-edge inner-shell spectrum of metal-free tetraphenylporphyrin, the energy difference between the LUMO and LUMO+1 peaks was above 2 eV owing to the detachment of central metal ions. Such a separation of the LUMO and LUMO+1 peaks was not observed in the present measurements. This fact also supports the interpretation that the Mg^{2+} ion was not desorbed from Chl-a with the radiation-induced effect during the successive scans of the N K-edge XAS measurements.

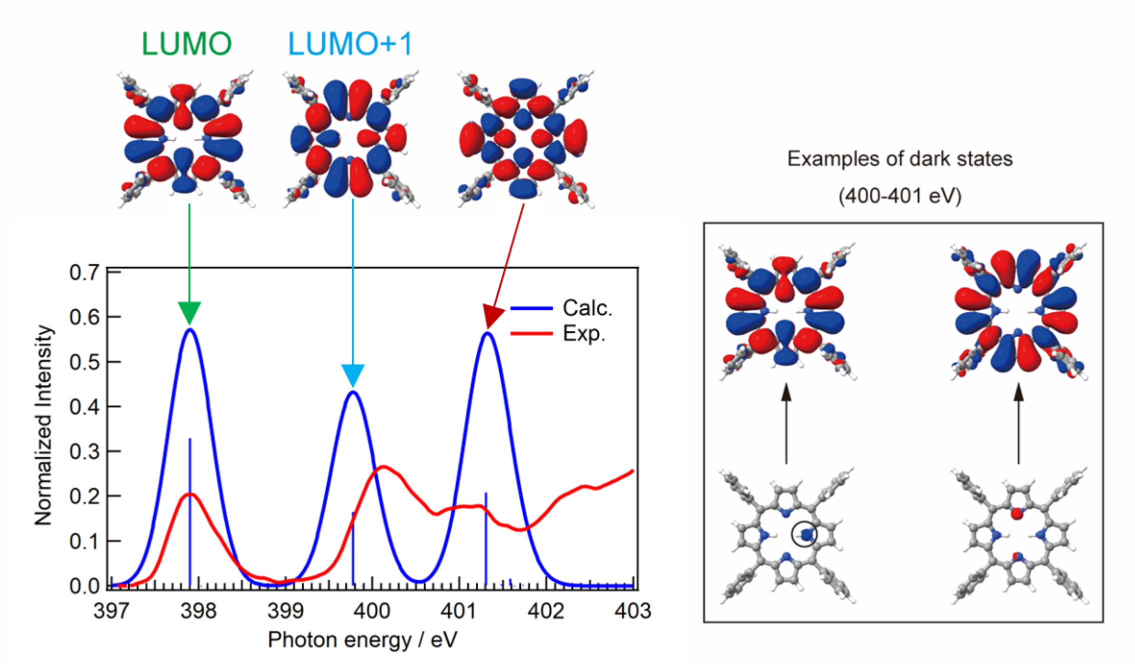

Figure S5. The calculated N K-edge inner-shell spectrum of tetraphenylporphyrin with no central metal, together with the experimental spectrum of solid tetraphenylporphyrin.

References

(1) Tsuru, S.; Vidal, M. L.; Pápai, M.; Krylov, A. I.; Møller, K. B.; Coriani, S. An Assessment of Different Electronic Structure Approaches for Modeling Time-Resolved X-ray Absorption Spectroscopy. *Struct. Dyn.* **2021,** *8*, 024101.

(2) Büchner, R.; Fondell, M.; Haverkamp, R.; Pietzsch, A.; Vaz da Cruz, V.; Föhlisch, A. The Porphyrin Center as a Regulator for Metal-Ligand Covalency and π Hybridization in the Entire Molecule. *Phys. Chem. Chem. Phys.* **2021,** *23*, 24765-24772.
